# Measuring Subjective Wellbeing in a School Context: A Polish Version of the Student Subjective Wellbeing Questionnaire

**DOI:** 10.1007/s12310-022-09546-x

**Published:** 2022-09-23

**Authors:** Magdalena Zadworna, Karolina Kossakowska, Tyler L. Renshaw

**Affiliations:** 1 Faculty of Educational Sciences, Institute of Psychology, University of Lodz, Rodziny Scheiblerów Avenue 2, 90-001 Lodz, Poland; 2 Psychology Department, Utah State University, 2810 Old Main Hill, Logan, UT 84322 USA

**Keywords:** Student subjective wellbeing, School, Assessment, Measurement

## Abstract

The Student Subjective Wellbeing Questionnaire is a measure designed to assess adolescents’ subjective wellbeing at school. The article presents our work toward adapting the SSWQ to the Polish cultural context. The Polish translation of the SSWQ, the KIDSCREEN–27, and the State-Trait Anxiety Inventory for Children (STAIC) were administered to 818 students aged 10–16 years, who were in Grades 5–8 of elementary school. As a result of a language adaptation process, a 16-item questionnaire was created, comprising four subscales, like the original version: School Connectedness, Joy of Learning, Educational Purpose, and Academic Efficacy. Confirmatory factor analysis demonstrated that both the SSWQ-PL first-order measurement model, which consisted of the above four fully correlated factors, and its second-order measurement model, which structured these four first-order factors as indicators of one second-order factor (i.e., student subjective wellbeing), showed good data–model fit and high internal consistency with the present sample. Cronbach’s alpha for the overall score was .87 and *H* coefficient was .94. The Pearson product-moment correlation coefficient between the total SSWQ-PL scores at two time points was.88 (*p* < *.*01), which suggests that the SSWQ-PL is reliable over time. Results showed that the SSWQ-PL had appropriate convergent and divergent validity with scores from the KIDSCREEN-27 and STAIC, which means it can be a useful measure to assess students’ subjective wellbeing in school counseling.

## Introduction

The concern to ensure an adequate level of wellbeing for all citizens, and above all for children and adolescents, has special social significance. Understanding the needs and rights of the youngest generation is crucial because investing in children means investing in the society that they will create and contribute to in the future (WHO, 2020a). The meta-construct *wellbeing* encompasses different aspects of successful and healthy living. Mental health screening in school settings can target subjective wellbeing (Renshaw et al., 2015), which is a person’s cognitive and affective evaluations of his or her life, including both emotional reactions and cognitive judgments of satisfaction (Diener et al., 2012). In this study, we use the term subjective wellbeing to refer to self-reported personally or socially desirable private behavior (i.e., thoughts and feelings) or public behavior (i.e., overt actions) (Renshaw, 2016).

The effort to measure and monitor children’s and adolescents’ subjective wellbeing and its indicators is not new. However, recent years have brought new and growing attention to the field. Young people in the WHO European Region enjoy better health and development than ever before, but are failing to achieve their full potential. Important results are provided every four years by the widely administered Health Behavior of School-Aged Children (HBSC) survey (WHO, 2020b), which monitors the state of health, wellbeing, the social environment and contributes to preserving the health of European youth aged 11–15. Although the general subjective wellbeing of young people seems to be relatively high (with an overall life satisfaction score of 7.8 out of 10), it is influenced by self-image, relationships with parents, moods and emotions, school environment, and socioeconomic factors (Nagata, 2020). Boys, younger adolescents, and respondents from richer families report better mental health. Other determinants of life satisfaction are health behaviors, support and positive family communication, and adolescent loneliness levels (Currie & Morgan, 2020).

Young peoples’ wellbeing is strongly influenced by the school context. Data concerning young people’s perception of the school environment, school satisfaction, school achievement, and school climate seem to be alarming. Adolescents feel pressured by schoolwork. In most countries/regions, school experience worsens with age: school satisfaction and support from teachers and classmates decline, and schoolwork pressure increases. Specifically, the latest research reports from Poland showed that students’ attitude toward school and perceived school-related social support deteriorates, while the percentage of students experiencing high levels of stress at school increases (Mazur & Małkowska-Szkutnik, 2018). Other studies on the subjective wellbeing of children and adolescents in Poland have yielded similar results. Despite a relatively high general level of life satisfaction, school functioning is often assessed as the lowest level (Kossakowska & Zadworna, 2019; Strózik et al., 2016). With requirements, workload, and continuous evolution with education system changes, Polish school is the greatest source of stress for young people (Sikora & Pisula, 2002).

This suggests the need for a more precise exploration of factors related to the school environment in Poland. Fostering adolescents’ mental health should start from the mental health screening in schools conducted with reliable measurement tools. The evidence in favor of screening for subjective wellbeing is primarily derived from findings showing meaningful relationships between measures of students subjective wellbeing and a variety of concurrent valued life outcomes (Arslan & Coşkun, 2020; Arslan & Renshaw, 2018). Student subjective wellbeing is a substantive predictor of antisocial and prosocial behavior, alcohol and tobacco use, psychological health problems, nutrition habits, school dropout, academic satisfaction, and school achievements. Therefore, student subjective wellbeing is an essential resource for improving youth academic functioning and psychological health. Children and adolescents spend a substantial amount of time in the school setting, which makes this setting a significant influence on their cognitive, social, and emotional development (Freeman et al., 2009; Rasmussen et al., 2005; Wang & Dishion, 2012). Positive school experience is considered a resource for health and quality of life, while negative school experience may constitute a risk factor, affecting both mental and physical health (Zadworna-Cieślak & Kossakowska, 2018).

Valid measurement is the first and indispensable step toward understanding the complexities of student wellbeing, which can inform school-based interventions and prevention programs (Gigantesco et al., 2019; Zadworna et al., 2020). To help improve the wellbeing of school-aged children and adolescents, therefore, it is extremely important for educators, caregivers, mental health professionals, and government leaders to intentionally consider the student subjective wellbeing domain—including such factors as school achievement, peer acceptance, connectedness, and sense of autonomy. Unfortunately, there is no available measure of student subjective wellbeing in Poland, even though there are tools for children and adolescents that assess global wellbeing or health-related quality of life. Adapted to or developed in the Polish context, those measures cover the most important domains of physical, psychological, and social wellbeing (Oleś, 2010). The school specific wellbeing domains, however, seem to be overlooked.

Working from the principles of contemporary test validation and adaptation (International Test Commission, 2017; Reeves & Marbach-Ad, 2016), the Student Subjective Wellbeing Questionnaire (SSWQ) was chosen for validation and adaptation to the Polish context. The SSWQ is a 16-item self-report instrument for assessing school-specific wellbeing. It measures overall subjective wellbeing and four more specific student wellbeing constructs, namely: *school connectedness*, defined as feeling cared for by and relating well to others at school; *joy of learning*, referring to the experience of positive emotions and cognitions during engagement in academic tasks; *educational purpose*, meaning the appraisal of school and academic tasks as important and meaningful; and *academic efficacy*, defined as appraising one’s academic behavior as effectively meeting environmental demands (Renshaw et al., 2015). These four student wellbeing constructs were identified from the literature and the preexisting measurement research targeting young people’s self-perceptions of their healthy and successful functioning at school.

The initial development and validation of the SSWQ was carried out on a sample of 1,002 students living in the southern region of the USA, mostly identified as Black/African American in Grades 6 to 8 (Renshaw et al., 2015). The latent structure of the measure was determined via exploratory and confirmatory factor analyses, which showed that the hypothesized measurement model adequately fit the data. The overall scale and each four-item subscale had robust item loadings and good internal consistency reliability. The four scales and the latent constructs they represent are psychometrically sound across genders and convergent with other subjective wellbeing measures. Overall, the psychometric properties of the SSWQ are satisfactory, and the tool appears to be a structurally valid measure of student subjective wellbeing.

A replication study confirmed the technical adequacy of the SSWQ with a demographically similar sample of adolescents in Grades 6 to 7 (*N* = 436). Results also indicated relationships between subjective wellbeing and young people’s self-reported academic achievement, cumulative risks, and cumulative assets, confirming the usefulness of the SSWQ for school psychological research and practice (Renshaw, 2015). Further validation of the SSWQ with a sample of urban middle-school students (Grades 5–8, *N* = 335) confirmed its higher-order measurement model. The study also demonstrated that both first-order and second-order factors had substantive effects for predicting several school-reported outcomes—including students’ standardized test performance in math and language arts as well as behavioral conduct at school—although first-order factors were more robust predictors overall (Renshaw & Chenier, 2018).

The internal structure of the SSWQ has also been analyzed with a sample of 548 Turkish adolescents in Grades 9 to 12, aged 14–19, in public high schools in an urban city. The study generalized the structural validity of the SSWQ’s higher-order measurement model with a demographically different sample, while also demonstrating that the second-order subjective wellbeing factor was a strong predictor of several school-specific wellbeing factors, including motivation, attitudes, and self-perceptions (Renshaw & Arslan, 2016). Another study with Turkish adolescents (*N* = 374) revealed that the SSWQ’s first-order measurement model, which consisted of four fully correlated factors (i.e., joy of learning, school connectedness, academic efficacy, and educational purpose), and the second-order measurement model, which structured these four first-order factors as indicators of one second-order factor, both showed good data–model fit, high internal reliability, and strong predictive power in accounting for the variance in the problem behaviors (Arslan & Renshaw, 2018). Validity evidence from these studies suggested the instrument may be appropriate for use as a schoolwide screener, progress monitoring tool, or a general outcome measure. To date, there has been no other attempts to validate the SSWQ in other countries. Further research is warranted to investigate the psychometric properties of the SSWQ with participants from diverse populations.

### The Present Study

The aim of the present study was to create a Polish language adaptation of the SSWQ and then evaluate the technical adequacy of this version of the measure (SSWQ-PL) within a Polish school context. The following research questions were investigated with an adolescent sample:

1. What is the structural validity, internal consistency, and test–retest reliability of the SSWQ-PL?
2. What are the mean-level differences in school-specific subjective wellbeing across youth with different demographic characteristics (i.e., gender, grade level)?
3. What evidence is there for the convergent and divergent validity of the SSWQ-PL in terms of associations with health-related quality of life indicators as measured by the KIDSCREEN-27 (i.e., physical wellbeing, psychological wellbeing, autonomy and parent relations, peer social support, and functioning at school environment) and with anxiety as measured by State-Trait Anxiety Inventory for Children (STAIC)?

It was expected that SSWQ-PL subscale scores would have high inter-item correlations. Since the internal structure identical to the original SSWQ measurement model was obtained in research with a Turkish sample (Arslan & Renshaw, 2018; Renshaw & Arslan, 2016), a four-factor latent structure corresponding to the original version was expected to replicate with a Polish language version and sample. We predicted that the internal consistency reliability of the SSWQ-PL would achieve at least minimal acceptability when assessed for the full sample and when differentiated by gender and grade level. We also expected high test–retest reliability (absolute stability) over an interval of three weeks. Scores on the SSWQ-PL were expected to be significantly positively associated with KIDSCREEN-27 scores and negatively correlated with STAIC scores, and these associations were expected to be similar across genders and grade levels.

## Method

### Procedure and Data Collection

A cross-sectional design with purposive sampling was chosen for the study, which involved recruiting male and female adolescents aged 10 to 16 years, who were students attending nine elementary schools in central Poland. Data were collected between January 2018 and February 2020. First, we contacted eligible schools to explain the purpose of the study to the school heads. Overall, 9 out of 15 primary schools located in an urban area in central Poland expressed interest in the study. After obtaining agreement from the school head, members of our research team or a designated school counselor met with the students’ parents to present the purpose and course of the study and to obtain their informed consent to students’ participation. All parents’ consent was obtained in each of the selected schools.

The study was conducted by members of the research team. The research was carried out in groups during regular class time. The respondents were informed about the purpose of the research and instructed on how to complete the tests; they were also informed that participation in the study was anonymous and voluntary and that it is not mandatory. All students gave their verbal informed consent. After three weeks, as suggested time for measurement of attitudes stability, (Jankowski & Zajenkowski, 2009; Nunnally & Ator, 1972), a re-measurement was carried out using SSWQ only with the participation of students attending two selected classes. The classes for a re-test were selected on the basis of two criteria: the consent of the school head and the teachers to re-use the class time for the questionnaire study, and the same or the closest students’ attendance to the day of the first measurement. Research team member re-informed students about the purpose of the research and instructed on how to complete the test; they were informed again that participation in the study was anonymous and voluntary and not mandatory. All students gave their verbal informed consent.

The inclusion criteria for student participants were as follows: between 10 and 16 years old, being an elementary school student (in Grades 5 to 8), parental signed and students’ verbal informed consent to participate in the study. The exclusion criteria were age over 16 and a lack of parental and student’s own informed consent. Each student who met the eligibility criteria was asked to complete a set of self-assessment questionnaires administered by paper and pencil. Overall, students from 30 classes took part in the study, with 3 classes representing different grades selected in each school. The number of students in a class ranged from 26–30. Of the 890 questionnaires distributed, 72 returned incomplete or incorrectly completed, which means that results from 818 students were included in the analysis. The overall student response rate was 91.9%.

The research was conducted in accordance with the Helsinki Declaration of Human Rights (WMA, 2013). The study protocol was approved by the Research Ethics Board at the University of Lodz. We obtained agreement from the head of each school where the study was to be conducted and consents from the parents of students. Both students and their parents were informed that the results of the study would only be used for research purposes, that participation was anonymous and voluntary, and that they could withdraw at any time without penalty.

### Polish Language Adaptation of the SSWQ

The original English version of the SSWQ (Renshaw & Chenier, 2018; Renshaw et al., 2015; Renshaw, 2015, 2018a) was translated into Polish. We followed the guidelines for test adaptation issued by the International Test Commission (2017). In the first stage, two independent bilingual translators translated the original statements from English into Polish (forward translation). Next, a team of competent judges was formed, consisting of five teachers (represented teachers from 4–8 secondary school grades), and five psychologists (two of whom were a researcher in the field of school psychology and three working as school psychologists). This selection of judges was intended to provide a diverse set of views about school mental health and subjective wellbeing. The judges assessed whether the statements in the current version were semantically appropriate and understandable in the Polish cultural context. They discussed Polish translation and reached a consensus on discrepancies. After minor revisions, the statements were translated back into English (back-translation). In the next stage, back translation was carried out by a third independent translator who was a native English teacher and translator. The semantic equivalence and validity of the two versions was therefore ensured by three other experts: a translator, two psychologists, and a teacher. The translated version of the tool, compared to the original, did not contain any significant changes, including the order and content of the items and instruction. The final Polish version SSWQ-PL was established, which was used in the presented study.

### Participants

The study included 818 Polish elementary school students in Grades 5–8. The students’ mean age was 13.04 years (*SD* = 1.45; range: 10–16). The group consisted of 417 boys (51%) and 401 girls (49%). All participants were White/Caucasians. In the final sample, 31.5% were fifth graders (*n* = 258), 18.3% were sixth graders (*n* = 150), 20.5% were seventh graders (*n* = 168), and 29.6% were eighth graders (*n* = 242).

As shown in Table 1, the age difference between students at the same level of education varies from one year to 4–5 years. This may be due to several reasons: the date of birth of individual children, grade repetition (due to unfulfilled educational requirements, but also, for example, due to diseases that prevented the child from completing compulsory schooling within the statutory deadline), or the changes and education reforms introduced in Poland in the years including the study period. The discrepancy in the age of students within a given level of education should be mainly explained by the fact that some students who entered school before the introduction of the latest reform entered the first grade as 6-year-olds, whereas the education reform of 2019 moved the beginning of compulsory schooling to the age of 7. Detailed characteristics of the study sample are presented in Table 1.

**Table 1:** Participants’ demographic characteristics

Variable					*n*	%
Gender						
	Female				401	49,0
	Male				417	51,0
Grade						
		Age	*n*	*%*		
	5th	10	8	3.1	258	31.5
		11	150	58.1		
		12	93	36.0		
		13	6	2.3		
		14	1	.4		
	6th	11	6	4.0	150	18.3
		12	53	35.3		
		13	83	55.3		
		14	8	5.3		
	7th	12	10	6.0	168	20.5
		13	54	32.1		
		14	102	60.7		
		15	1	.6		
		16	1	.6		
	8th	14	79	32.6	242	29.6
		15	5	2.1		
		15	155	64.0		
		16	3	1.2		

### Measures

#### Student Subjective Wellbeing Questionnaire-polish version (SSWQ-PL)

The Polish version (like the original version) of the SSWQ consists of four subscales, namely: the School Connectedness Scale (SCS), the Joy of Learning Scale (JLS), the Educational Purpose Scale (EPS), and the Academic Efficacy Scale (AES). The Student Subjective Wellbeing Questionnaire yields a total score that is computed by summing all subscale scores. The Polish version of the SSWQ consisted of the same number of items (16), intended to represent the same four constructs and rated on the same 4-point response scale (1 = *almost never*, 2 = *sometimes*, 3 = *often*, 4 = *almost always*) as the items of the English version. Cronbach’s alpha coefficients for the scales in the original study varied from 0.72–0.97 for SCS, from 0.74–0.76 for JLS, from 0.72–0.73, for EPS, from 0.78– 0.86 for AES, and from 0.86–0.88 for the overall scale (SSWQ total).

#### The KIDSCREEN-27 Health-related Quality of Life Questionnaire for Children and Adolescents

We used the KIDSCREEN-27 to assess health-related quality of life (HRQL) in childhood and adolescence (age 8–18; (Ravens-Sieberer et al., 2007). The tool measures overall HRQL and its five domain-specific components: (1) Physical Wellbeing: the level of physical activity and health complaints (5 items); (2) Psychological Wellbeing: emotions and satisfaction with life (7 items); (3) Autonomy and Parent Relations: perceived autonomy, finances, and the quality of support from parents (7 items); (4) Social Support and Peers: the quality of interaction with and perceived support from friends (4 items); and (5) School Environment: perceived cognitive capacity and feelings about school and teacher relations (4 items). Every factor comprises at least four questions. Respondents give their answers on a 5-point Likert scale (ranging from *never* to *always*); the higher the scores, the better the HRQL. The 27-items version was developed as a short version of the KIDSCREEN-52 and has good psychometric properties with minimum information loss compared to the full version (The KIDSCREEN Group Europe, 2006). A psychometric analysis of the Polish version of research questionnaires concerning the quality of children’s and teenagers’ life was carried out by the research team of the Institute of Mother and Child in Warsaw (Mazur et al., 2008). The use of the instrument was registered in accordance with the KIDSCREEN Group instruction, and permission to use the instrument was granted by the KIDSCREEN Group. The reliability of the measure in the current sample was excellent, as Cronbach’s alpha for the total score was 0.90.

#### The State–Trait Anxiety Inventory for Children (STAIC)

The State–Trait Anxiety Inventory for Children (STAIC) was developed by Spielberger (1973). The inventory measures state anxiety, understood as a situationally conditioned and transient state of an individual, and trait anxiety, understood as a relatively constant personality trait. The STAIC includes two independent subscales: The X-1 scale is used to measure state anxiety, whereas X-2 is used to measure trait anxiety. Each of the scales consists of 20 items, summing to 40 total items. The measure is one of the most frequently used self-report instruments for evaluating anxiety in children and adolescents (aged 10–14). The STAIC has strong internal consistency and acceptable construct validity. In our study, we used the Polish adaptation of the measure (Jaworowska, 2005). Its reliability coefficients were very good in the current study, as Cronbach’s alpha was 0.89 for the State Anxiety scale and 0.88 for the Trait Anxiety scale.

### Data Analysis

The data were analyzed using the Statistical Package for the Social Sciences (SPSS) version 25, and structural equation modeling (SEM) was performed using AMOS SPSS version 25. In the final database, response frequency analyses of the SSWQ-PL items indicated missing data ranges of 0.5– 1.5%. To handle missing data, the pairwise deletion method was used for all analyses conducted with SPSS. Preliminary analyses included descriptive statistics and checking the normality of the measures of interest. The distributional properties were considered adequate when the absolute value of skewness and kurtosis were lower than 2 (George & Mallery, 2016).

To examine structural validity, we analyzed the factorial validity of the measure. We performed a confirmatory factor analysis (CFA) using the maximum likelihood (ML) method to investigate the latent structure of the Polish version of the SSWQ. General assumptions of CFA include linearity, continuity of variables, independence of observations, and sufficient sample size (*N* > 200). ML is the most frequently used method, recommended for large samples and normal distribution of variables (or slight deviations from the normal distribution). To evaluate model validity evidence, we considered data–model fit statistics in conjunction with factor loadings and other parameter estimates (Mueller & Hancock, 2011). To examine the goodness of data–model fit, we used a combination of absolute, parsimonious, and incremental fit indices. Tucker–Lewis Index (TLI) and comparative fit index (CFI) values between 0.90–0.95 and root mean square error of approximation (RMSEA) values (with a 90% confidence interval) between 0.05 and 0.08 were interpreted as indicating adequate data–model fit, while TLI and CFI values > 0.95 and RMSEA values < 0.05 were considered indicative of good data–model fit (Kenny, 2020). As regards factor loadings, values higher than 0.50 were considered to be strong, as they accounted for more than 25% of the variance extracted from each item by the latent factor.

Internal consistency was calculated using the standardized Cronbach’s alpha coefficient (α), which was considered adequate when *α* ≥ 0.70 (Nunnally, 1979; Streiner et al., 2015), and using the Guttman split-half coefficient (*λ*), regarded as adequate when *λ* > 0.60 (Guttman, 1945). Latent construct reliability was also established and coefficients (H) ≥ 0.70 were considered desirable (Mueller & Hancock, 2011). Absolute stability—test–retest reliability—was assessed over a three-week interval using Pearson product-moment correlations and intraclass correlation coefficients (ICC) in a sample of 61 students (attending to two chosen classes).

In order to examine if there were differences in the levels of school wellbeing and its dimensions across genders and grade levels, we used the independent *t*-test and one-way analysis of variance with Tukey’s post hoc tests. To evaluate the effect size for gender comparisons, we calculated Cohen’s *d* coefficient. According to Cohen’s guidelines (Cohen, 2013), values of *d* equal to 0.2, 0.5, and 0.8 correspond to small, medium, and large effects, respectively. We also calculated partial eta-squared (*η*_*p*_^2^) to assess the effect size for grade level comparisons. The suggested norms for partial eta-squared are as follows: small *η*^2^ = 0.01, medium *η*^2^ = 0.06, large *η*^2^ = 0.14 (Cohen, 2013).

Convergent and divergent validity of the SSWQ-PL scores with other concurrent indicators was assessed with Pearson’s correlational analysis (*r*). For concurrent validity, we estimated the correlation between SSWQ-PL and KIDSCREEN-27 scores. For divergent validity, we computed correlations between SSWQ-PL scores and the scores on the two subscales of the STAIC.

In order to establish the sample size for *t*-tests, ANOVA, and correlation analysis, we performed a a-priori power analysis using G*Power 3.1 software (Faul et al., 2007). With a medium effect size (*α* = 0.05, a standard power level of 0.95), a required minimum sample size for all types of analyses was attained (i.e., 176 participants for *t*-test, 138 for correlation, 305 for ANOVA with 4 groups). Post hoc power analysis revealed an excellent power close to 1 for all analyses conducted in this study group. The recommended minimum sample size for SEM was also reached (Kenny, 2020; Kline, 2015).

## Results

### Descriptive Statistics

Results concerning the item distribution of SSWQ-PL showed that the Kolmogorov–Smirnov test results were significant, indicating non-normality. However, departures from normality were mild for every item. Skewness values for all variables do not exceed absolute 2, which means that these distributions are symmetrical and can be regarded as approximately normal. Also, based on the central limit theorem, the size of the study group makes it possible to assume normal distributions (Fischer, 2011). Detailed descriptive statistics for SSWQ items is presented in Table 2.

**Table 2:** Descriptive statistics and item-total correlations of SSWQ-PL items

	*M*	*Me*	*SD*	*Sk*	*Kurt*	*K-S*	Item-total correlations	Cronbach’s *α* when item deleted
Item No								
SSWQ1	2.51	2.00	.79	.30	−.45	.29*	.54	.86
SSWQ2	2.41	2.00	1.03	.18	−1.10	.22*	.44	.86
SSWQ3	2.34	2.00	.99	.27	−.93	.24*	.53	.86
SSWQ4	2.86	3.00	.89	−.19	−.92	.20*	.47	.86
SSWQ5	2.39	2.00	.85	.25	−.51	.27*	.61	.85
SSWQ6	2.44	2.00	1.06	.14	−1.19	.21*	.42	.86
SSWQ7	2.85	3.00	.91	−.30	−.78	.22*	.51	.86
SSWQ8	2.87	3.00	.91	−.26	−.92	.19*	.50	.86
SSWQ9	2.43	2.00	1.01	.19	−1.04	.23*	.48	.86
SSWQ10	2.13	2.00	.94	.48	−.65	.25*	.41	.86
SSWQ11	2.86	3.00	.92	−.35	−.76	.22*	.64	.85
SSWQ12	2.85	3.00	.86	−.18	−.81	.21*	.53	.86
SSWQ13	2.10	2.00	.86	.50	−.34	.27*	.60	.85
SSWQ14	2.61	3.00	.97	−.11	−.96	.21*	.37	.86
SSWQ15	2.70	2.00	1.03	−.13	−1.17	.20*	.49	.86
SSWQ16	2.92	3.00	.86	−.22	−.87	.21*	.49	.86

*M* – mean, *Me* – median, *SD* – standard deviation, *Sk* – skewness, *Kurt* – kurtosis, *K−S* – Kolmogorov−Smirnov’s test of normality, * indicates *p* < .05

### Structural Validity

We performed a confirmatory factor analysis (CFA) using the maximum likelihood method to investigate the latent structure of the Polish version of the SSWQ. The assumptions of ML method were met, including a large sample size and observed indicators following a continuous and multivariate normal distribution. The results of the initial CFA, which structured the 16 items in the Polish version of the SSWQ as indicators of four fully correlated first-order latent factors (school connectedness, joy of learning, educational purpose, and academic efficacy), indicated adequate data–model fit (*χ*^2^ = 401.614, *df* = 98, *p* < 0.001, CFI = 0.932, TLI = 0.906, NFI = 0.913, RMESA = 0.062, 90% CI [0.055, 0.068]). The analysis revealed that the chosen theoretical construct had a significant influence on the variability of scores for specific items (see Table A1 in Appendix). The model was characterized by robust factor loadings for each latent construct, with beta coefficients ranging from 0.54–0.84, and *R*^2^ coefficients ranging from 0.29 to 0.71, *p* < 0.001.

**Fig. 1:**
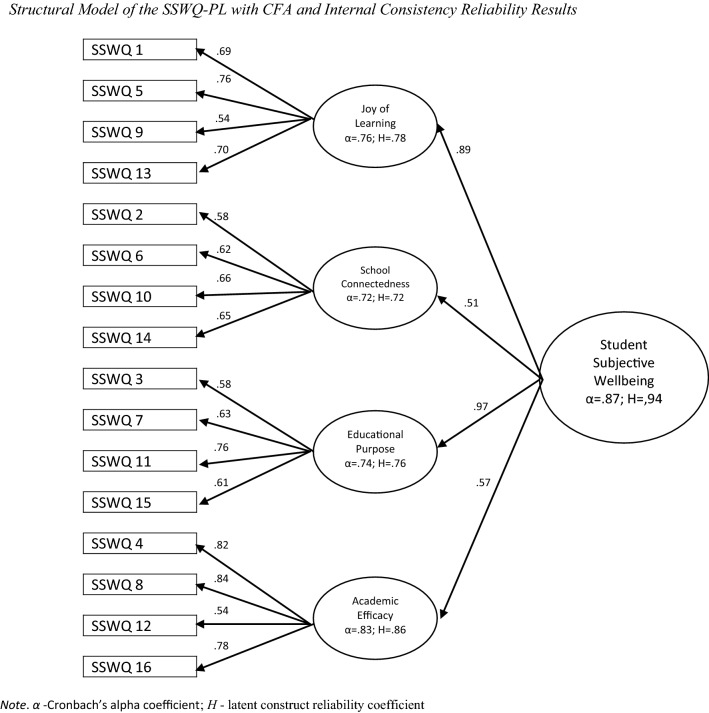
Structural model of the SSWQ-PL with CFA and internal consistency reliability results. *α* -Cronbach’s alpha coefficient; *H*—latent construct reliability coefficient

Model 2, which extended Model 1 by structuring the four first-order latent factors as effect indicators of a single second-order latent factor (i.e., student subjective wellbeing), also yielded an adequate data–model fit (*χ*^2^ = 415.504, *df* = 100, *p* < 0.001, CFI = 0.929, TLI = 0.904, NFI = 0.910, RMESA = 0.062, 90% CI [0.056, 0.068]). Findings from both models indicated that all factor loadings, for all first-order latent variables and for the second-order latent trait, were significant and robust in the hypothesized direction (Fig. 1). Further analysis indicated correlations between certain dimensions (see Table 3). The analysis found the four-factor model to be well fitted to the data, and the test measurements were a strong and accurate expression of the latent variables. Adequate-to-strong latent construct reliabilities were also established for all factors in the model ((JL H = 0.78, SC H = 0.82, EP H = 0.76, AE H = 0.86), and for higher-order factor of general student wellbeing (H = 0.94).

**Table 3:** Intercorrelations among the SSWQ-PL scales

Scale	1	2	3	4	5	*M*	*SD*
1. JLS	1					9.42	2.68
2. SCS	.41**	1				9.58	2.96
3. EPS	.65**	.37**	1			10.76	2.89
4. AES	.42**	.24**	.46**	1		11.51	2.86
5. SSWQ Total	.81**	.68**	.82**	.70**	1	41.39	8.60

^**^ indicates *p* < .01. JLS—Joy of Learning Scale; SCS—School Connectedness Scale; APD—Educational Purpose Scale; AES—Academic Efficacy Scale

### Internal Consistency Reliability

First, we assessed the internal consistency reliability of all the items of the scale using Cronbach’s alpha (*α*) internal consistency coefficient, which is considered adequate when *α* ≥ 0.70. Cronbach’s α showed good internal consistency for the total score of SSWQ-PL (*α* = 0.87) and for the four factors: JL (four items) *α* = 0.76, SC (four items) *α* = 0.72, EP (four items) *α* = 0.74, and AE (four items) *α* = 0.83. Inter-item correlations of the 16 items ranged from 0.38–0.64 (see Table 2); this means there was no reason to remove any items, which is recommended when inter-item correlations are lower than 0.30 and are therefore considered poor (Tabachnick & Fidell, 2019).

Split-half reliability was also applied. The 16 items were split pseudorandomly into two halves, with eight items in each half. The mean (± *SD*) scores of each half were found to be similar (20.7 ± 4.5 vs. 20.6 ± 4.5). The correlation between the two halves was 0.80, and the Guttman split-half coefficient was 0.87, which is also acceptable. Finally, we examined the internal consistency reliability for SSWQ-PL total score by gender and grade level. All Cronbach’s alphas were acceptable for both genders (*α*_boys_ = 0.87; *α*_girls_ = 0.86) and grade levels (*α*_5th_ = 0.86; *α*_6ht_ = 0.89; *α*_7th_ = 0.88; *α*_8th_ = 0.85).

### Test–Retest Reliability

Three weeks after initial data collection, 61 5th and 6th grade students were re-tested. However, the optimal time interval for evaluating test–retest reliability should be determined individually for each examination, and three weeks is the most common interval for attitudes measuring (Streiner et al., 2015). First, we computed the Pearson correlation coefficient between SSWQ-PL total scores for first (T1) and second (T2) measurements; its value was *r*_*tt*_ = 0.88 (*p* < 0.01). Then, we computed the intraclass correlation coefficient (*ICC*) and found its value to be 0.85 with a 95% confidence interval between 0.71–0.92, indicating excellent interrater reliability. The results of the analyses for SSWQ subscales were as follows: for JLS: *r*_*tt*_ = 0.87, *p* < 0.01, *ICC* = 0.84, 95% CI [0.72, 0.91]; for SCS: *r*_*tt*_ = 0.89, *p* < 0.01, *ICC* = 0.87, 95% CI [0.78, 0.92]; for EPS: *r*_*tt*_ = 0.95, *p* < 0.01, *ICC* = 0.93, 95% CI [0.86, 0.96]; for AES: *r*_*tt*_ = 0.92, *p* < 0.01, *ICC* = 0.88, 95% CI [0.76, 0.94].

### SSWQ Scores by Demographic Characteristics

SSWQ-PL total and subscale scores were correlated with age and compared between subgroups distinguished according to gender and grade level. Participants’ age was significantly negatively correlated with scores on the Joy of Learning (*r* =  − 0.071, *p* = 0.044) and Educational Purposes (*r* =  − 0.192, *p* < 0.01) subscales and with SSWQ total scores (*r* =  − 0.104, *p* = 0.004). However, the correlations were very weak (see Table 4). Compared to boys, girls scored significantly higher on Joy of Learning (*t*_(799)_ = 5.044, *p* < 0.001), Educational Purpose (*t*_(805)_ = 3.519, *p* < 0.001), Academic Efficacy (*t*_(801)_ = 4.795, *p* < 0.001), and total SSWQ (*t*_(768)_ = 5.482, *p* < 0.001). There were no gender differences in School Connectedness (*t*_(796)_ = 1.076, *p* < 0.282). There were also statistically significant differences in Educational Purpose (*F*_(3, 803)_ = 11.333, *p* < 0.001), Academic Efficacy (*F*_(3, 799)_ = 4.385, *p* = 0.005), and total SSWQ (*F*_(3, 766)_ = 3.077, *p* = 0.027) depending on grade level. Educational Purpose scores were significantly higher in fifth grade level students (*M* = 11.54, *SD* = 2.9) in comparison to sixth (*M* = 10.43, *SD* = 3.0; *p* < 0.01) and eighth (*M* = 10.10, *SD* = 2.8, *p* < 0.001) grades. Academic Efficacy scores were significantly lower in sixth grade level students (*M* = 10.94, *SD* = 2.8) than in eighth-graders (*M* = 11.95, *SD* = 2.9, *p* < 0.01). Finally, the total SSWQ scores were significantly higher in fifth grade level students (*M* = 42.80, *SD* = 8.3) than in those representing eighth grade level (*M* = 40.75, *SD* = 8.3, *p* < 0.05) (see Table 4).

**Table 4:** SSWQ scores by demographic characteristics

		Joy of learning (JLS)	School Connectedness (SCS)	Educational Purpose (EPS)	Academic Efficacy (AES)	SSWQ total
Age		−.07^*^	−.04	−.19^**^	.04	−.10^**^
Gender						
	Girls	9.9 (2.7)	9.7 (3.1)	11.1 (2.7)	12.0 (2.8)	43.1 (8.4)
	Boys	8.9 (2.6)	9.5 (2.8)	10.4 (3.0)	11.0 (2.8)	39.8 (8.5)
	t-test	5.04***	1.08	3.52***	4.98***	5.48***
	Cohen's *d*	.38 [.23−.52]	*NA*	.25 [.11−.38]	.36 [.22−.49]	.39 [.25−.53]
Grade level						
	5th	9.65 (2.6)	9.72 (3.0)	11.54 (2.9)	11.59 (2.9)	42.80 (8.3)
	6th	9.48 (2.6)	9.66 (2.9)	10.43 (3.0)	10.94 (2.8)	40.80 (9.2)
	7th	9.36 (2.6)	9.35 (3.0)	10.81 (2.8)	11.27 (2.8)	40.77 (8.8)
	8th	9.18 (2.7)	9.57 (3.0)	10.10 (2.8)	11.95 (2.9)	40.75 (8.3)
	F-test	1.31	.55	11.33***^1^	4.39**^2^	3.08*^3^
	*η*_*p*_^*2*^	*NA*	*NA*	.04 [.02−.06]	.02 [.00−.03]	.01 [.00−.02]

Figures for age were Pearson’s correlation coefficients

Standard errors are in brackets

^*^ indicates *p* < .05; ** indicates *p* < .01; *** indicates *p* < .001. *NA* indicates ‘not applicable’

Cohen's *d –* effect size for gender differences with 95% CI in brackets

*η*_*p*_^*2*^ – partial eta square effect size for grade level differences with 90% CI in brackets

*Tukey’s post-hoc* analysis: ^1^ differences between 5 and 6th grade ( *p* < .01) and between 5 and 8th grade (*p* < .001); ^2^ differences between 6 and 8th grade ( *p* < .01); ^3^ differences between 5 and 8th grade ( *p* < .05)

### Convergent and Divergent Validity

We assessed convergent validity by correlating the SSWQ-PL total score with KIDSCREEN-27 subscale scores. All of the latter were found to correlate significantly (*p* < 0.01) with the SSWQ. The highest correlation was found between SSWQ total score and the School Environment subscale (*r* = 0.66; see Table 5). For divergent validity, we also computed correlation coefficients between SSWQ total score and STAIC subscales, measuring state and trait anxiety. Both of the latter were found to be significantly negatively correlated with SSWQ, but the correlation was higher for state anxiety (*r* =  − 0.26, *p* < 0.01) than for trait anxiety (*r* =  − 0.10, *p* < 0.05).

**Table 5:** Correlations of the SSWQ total score with KIDSCREEN-27 and STAIC scores

	Total sample				Girls			Boys		
Variable	*M*	*SD*	Range*	*r*	*M*	*SD*	*r*	*M*	*SD*	*r*
KIDSCREEN-27										
Physical Wellbeing	18.25	3.92	5–25	.24^**^	17.50	3.82	.32**	18.96	3.87	.24**
Psychological Wellbeing	24.03	5.78	7–35	.36^**^	23.89	5.92	.40**	24.16	5.66	.34**
Autonomy Parents	24.69	5.65	7–35	.34^**^	24.96	5.39	.34**	24.45	5.87	.32**
Peers Social Support	13.76	3.95	4–20	.23^**^	14.16	3.77	.22**	13.38	4.08	.22**
School Environment	12.27	3.33	4–20	.66^**^	12.65	3.32	.70**	11.91	3.31	.61**
General Index	93.39	16.25	51–131	.48^**^	93.20	15.97	.53**	93.58	16.54	.44**
STAIC										
State scale	34.30	9.63	20–74	−.26*	34.51	9.54	−.26**	34.10	9.72	−.27**
Trait scale	35.75	8.40	20–57	−.10*	37.31	7.94	−.21**	34.25	8.57	−.07

*M* – mean, *SD* – standard deviation

^*^ Range indicates possible score on each scale

^**^ indicates *p* < .01

KIDSCREEN-27 subscales and general index were expected to have positive correlations with the SSWQ total scores due to the convergent validity

STAIC subscales were expected to have negative correlations with the SSWQ total scores due to the divergent validity

Since we assumed that the relationships would be similar for the two genders and for all grade levels, we performed analogous analyses in the groups of boys and girls and in groups distinguished according to grade level. Both among boys and girls, the directions of correlations were as expected, and the strongest correlations were found between SSWQ total score and the School Environment subscale (*r* = 0.70 and *r* = 0.61 for girls and boys, respectively). Only the relationship between SSQW total score and the Trait Anxiety scale among boys was found to be statistically non-significant (*r* =  − 0.07, *p* = 0.23). Mean scores for the variables and the correlation coefficients between them are given in Table 5.

We performed a similar analysis to examine the convergent and divergent validity of the SSWQ across grade levels. The KIDSCREEN-27 subscale scores for all grades were found to correlate significantly (*p* < 0.01 or *p* < 0.05) with the SSWQ except with the Psychological Wellbeing scale (*r* = 0.16, *p* = 0.15) and the Peer and Social Support scale (*r* = 0.01, *p* = 0.94) among sixth-grade students. The STAIC State Anxiety scale was negatively correlated with the SSWQ for the fifth (*r* =  − 0.32, *p* < 0.01), seventh (*r* =  − 0.23, *p* < 0.01), and eighth (*r* =  − 0.28, *p* < 0.01) grade levels, while in the sixth grade this relationship turned out to be statistically non-significant (*r* =  − 0.17, *p* = 0.13). However, no statistically significant negative correlation was found between the SSWQ and the STAIC Trait Anxiety scale at any of the grade levels. Detailed analysis results are provided in Appendix (Table A2).

## Discussion

The present study created a Polish language adaptation of the SSWQ and then evaluated the technical adequacy of this version of the measure (SSWQ-PL) within a Polish school context. We also analyzed the mean-level differences in SSWQ-PL scores across demographic characteristic and tested convergent and divergent validity of the tool. The first research question concerned the structural validity, internal consistency, and test–retest reliability of the Polish version of SSWQ.

Based on the findings from the original SSWQ development study (Renshaw et al., 2015) and the replication study (Renshaw, 2015) conducted in USA, we expected our results to indicate that the Polish version of the SSWQ had a four-factor structure. The CFA showed that the fit indices of the adapted measure were excellent and corresponded with the original model of the SSWQ. Thus, the Polish version was found to be consistent with the factor structure of the original SSWQ. This seems to be particularly important because the Polish sample differed significantly from the original one—the educational system is different and the students participating in the Polish research were White Caucasians, while in the original and replication studies it was Black American students who were predominant. A similar factor structure of the SSWQ was also established in the Turkish sample (Arslan & Renshaw, 2018; Renshaw & Arslan, 2016). This model observed in previous studies was confirmed in a further validation study with another US sample (Renshaw & Chenier, 2018). Our study confirmed the robust psychometric properties of the SSWQ measurement model and provides evidence of its structural validity. While cultural and educational context differences exist between Polish, US, and Turkish adolescents, the results suggest the SSWQ’s measurement model may be relatively universal and therefore generalizable across languages and cultural contexts. Adequate-to-strong latent construct reliabilities were also established for all factors and for higher-order factor of general student wellbeing.

We expected that the internal consistency reliability of the SSWQ-PL in all analyses would reach at least the minimum acceptability level (α ≥ 0.70). The internal consistency of the SSWQ in original studies ranged between 0.86–0.88 for SSWQ total score (Renshaw, 2015; Renshaw et al., 2015). The current study yielded similar Cronbach's α values for the SSWQ-PL total score and for its subscale scores, with slightly lower values for the SC factor and slightly higher ones for the EP and AE factors, which attests to the adequate internal consistency of the Polish version. Internal consistency reliability differentiated by gender and grade level also was found to be acceptable in the Polish population (Cronbach’s α values ranged between 0.85–0.89). Good internal consistency was additionally confirmed by acceptable split-half reliability and item-total score correlations.

We also expected high test–retest reliability, which is another consistency criterion for time stability. Both Pearson’s correlation coefficient and the intraclass correlation coefficient (*ICC*) between the two scores were found to be statistically significant (*p* < 0.01) and indicated moderate to strong associations across time. It is worth noting that, to our knowledge, the temporal stability of the SSWQ has not been analyzed before and therefore no point of reference is available, but the results of the current study indicate that the measure is stable over time.

The second research question concerned the mean-level differences in SSWQ scores across demographic characteristic. As compared to boys, girls scored significantly higher in three domains (JLS, AES, AEF) and overall subjective wellbeing. SSWQ-PL total and subscale scores were weakly and negatively correlated with age. Students in the early grades had lower levels of wellbeing than those attending higher grades. A different result was obtained in the original study at the development of the measure, which revealed its measurement invariance across genders (Renshaw et al., 2015). However, the results of that study were contrary to previous findings, showing that elementary age female students had higher subjective wellbeing than males (Furlong et al., 2013). In the replication study (Renshaw, 2015), some gender differences were found—female students reported higher AEF than male students, but that there were no other notable differences in other first-order or second-order factors. For grade level, findings from the latent means analyses indicated that seventh-graders reported significantly lower subjective wellbeing, with negligible effect sizes; this was the case for JL, EP, and overall student subjective wellbeing. The authors suggest future research to replicate the latent structure of the SSWQ with more diverse samples of young people from different regions, in different grade levels (i.e., < Grades 6–8 <), and with different racial/ethnic backgrounds; the current study meets these criteria.

Some other research results indicate that older and male students may have lower levels of SWB at school than younger and female students (Kossakowska & Zadworna, 2019; Liu et al., 2016). Similar associations can be observed for the general wellbeing of adolescents—many results confirm that it decreases with age, but the findings regarding gender are sometimes contradictory (Ronen et al., 2016; Strózik et al., 2016). However, different tools were used in these studies and various groups were observed, which suggests the need for more detailed research using the SSWQ in the future, conducted in different populations and cultural contexts. Moreover, a replication of the Polish study could verify the demographic differences in the future.

The third research question concerned the convergent and divergent validity of the SSWQ-PL as shown by its associations with HRQL (in five domains: physical and psychological wellbeing, autonomy and parent relations, peer social support, and functioning at school environment) measured by the KIDSCREEN-27 and with anxiety measured by the STAIC. The obtained data revealed significant positive relationships between SSWQ-PL total score and HRQL. As expected, the associations were higher between students’ subjective wellbeing and the domain of functioning at school environment—that is, their perception of their own cognitive capacity and their feelings about school and teacher relations (*r*^2^ = 0.66, *p* < 0.01). The convergent validity of the SSWQ was also examined for gender and grade level invariance. Our results correspond with other research findings, which demonstrated positive relations of students subjective wellbeing to school prosociality and academic perseverance. The SSWQ was also convergent with other subjective wellbeing measures in the first validation study with a US sample (Renshaw et al., 2015).

Further replication and validation of the SSWQ revealed its substantive effects on several school-reported outcomes and various self-reported risks and assets (Renshaw, 2015; Renshaw & Chenier, 2018, 2019). Similar results were obtained in the Turkish sample, demonstrating convergent validity with several criterion variables that represented distinct yet related school-specific wellbeing constructs (Renshaw & Arslan, 2016). It is worth noting that in our research we have established the relations of the SSWQ to other types of wellbeing, not only that associated with school–such as physical wellbeing (i.e., the level of physical activity and health complaints) and psychological wellbeing (emotions and satisfaction with life). Moreover, perceived autonomy and quality of support from parents as well as the quality of interactions with peers were also related to students’ subjective wellbeing in our study.

Relationships between SSWQ-PL and STAIC subscales—state and trait of anxiety—showed the divergent validity of the measure. However, significant negative correlations were higher for state anxiety and for the general level of student wellbeing than for trait anxiety. State anxiety can be an indicator of subjective distress, a variable negatively related to student wellbeing, which corresponds with other research findings (Renshaw & Bolognino, 2016; Renshaw, 2018b). However, the associations of SSWQ score with trait anxiety are much weaker or even non-significant when the results are differentiated by age and gender. This suggests that student subjective wellbeing is related to emotional states rather than to more stable personality traits and dispositions. This fact has not been explored in other SSWQ studies until now and needs further investigation. We suspect there may possibly be a curvilinear relation between these variables, as teenagers who had moderate levels of trait anxiety are more successful at school than their peers with high or low anxiety (Duchesne et al., 2008). However, state anxiety can be also be induced through school pressure and testing situations, excessive demands, or lack of support, and therefore negatively affect school wellbeing and school outcomes (Newbegin & Owens, 1996).

While the relations between the SSWQ and HRQL are similar across genders and grades, in Grade 6 its associations with both HRQL and anxiety are very weak or not statistically significant. The reason may relate to specific rapid developmental changes that start in the early phase of adolescence, around the age of 12—typically during the 6th grade in the Polish educational system. Moreover, this is the time of decrease in the levels of SWB, which is usually the case from the age of 11–12 onwards (González-Carrasco et al., 2017). This decrease has been found in different countries and with diverse samples; it may be explained by the developmental changes occurring at these ages, concerning brain development, endocrinology, emotions, cognition, behavior, and interpersonal relationships (Goldbeck et al., 2007; Žukauskienė, 2014). Future applied research is needed to investigate issues connected with changes in student wellbeing throughout the course of development.

### Limitations and Directions for Future Research

Despite promising findings with important future implications, the current study also has several limitations that should be noted. First, this study was based on a subjective self-report questionnaire survey, and responses to items such as those in the SSWQ and other measures may be affected by social desirability bias. To prevent this bias in future research, we recommend extending the set of validation measures to include those that yield results other than self-report data. Data collected from interviews in conjunction, for example, with teacher and/or parent assessment of student wellbeing would help to minimize this mono-method bias. Therefore, in future validation studies, it is worth considering the use of the Student Wellbeing Teacher Report Scale (Roberson & Renshaw, 2019), which is a teacher-report measure targeting three context-specific indicators of youth wellbeing behavior: academic, social, and emotional wellbeing. Beyond rating scales, it would be valuable to include also other indicators of community involvement, peer relations sociometrics, school-reported indicators of academic grades, attendance, and dropout rates of the surveyed students. The associations of students’ subjective wellbeing with developmental phases, tasks, roles, and crises should also be investigated more closely, as our study has shown variance in SSWQ scores across grades, ages, and genders.

Second, the participants in current study were a convenience sample of students in the upper grades of elementary school, and therefore, the findings cannot be deemed representative of all upper-grade elementary school students in Poland. For this reason, the generalizability of our findings is limited in scope to demographically similar adolescents (i.e., fifth- to eight-graders, living in a large urban city, attending only public schools). A suggestion for a future SSWQ validation study with a Polish sample is to use random sampling techniques and recruit more diverse samples of students. Further investigations of measurement invariance should be conducted across genders, grade levels, types of school, and geographic locations.

Finally, the study period did not include the COVID-19 pandemic time and online education, as data were collected prior to the onset of schooling precautions related to this public health crisis. It could be especially interesting to investigate possible changes in student subjective wellbeing caused by the pandemic context. Relatedly, a longitudinal study is also warranted to observe changes in students’ wellbeing over a longer time period, such as over the course of one school year or even across multiple school years.

## Conclusion and Practical Implications

In the light of our findings, we suggest the SSWQ-PL can be considered a valid and reliable measure for evaluating adolescent students’ subjective wellbeing in the Polish population. The factor structure of the questionnaire is consistent across all studies, including those with US and Turkish samples. This strongly supports the generalizability of the four-factor model of school-specific subjective wellbeing indicators, and the latent structure underlying these constructs seems to be well verified. The results also demonstrate a new validity characteristic of the questionnaire, suggested by the authors of the measure in previous research—positive and theoretically consistent association between SSWQ-PL scores and scores on other multidimensional measures of subjective wellbeing. Moreover, our study is the first attempt to assess the time stability of the SSWQ, which was also recommended by the authors (Arslan & Renshaw, 2018; Renshaw, 2015). These results also suggest the need of mental health screening using the SSWQ in school contexts within different populations. General mean SSWQ score for Polish students observed in this study was 41.39, while the corresponding mean score for American students during the development of the original study was 49.54 (Renshaw et al., 2015). Future research is needed to explore this country-specific result.

The validity evidence supporting use of the SSWQ seems to be promising. Given that the measure is also free, publicly available online (Renshaw, 2018a), and brief (16 items), it is likely to be useful for practical purposes in school-based service delivery systems for promoting student social-emotional health and wellbeing. Our study provides further empirical evidence regarding the importance of research into student outcomes in school psychology, suggesting that assessments of school-specific subjective wellbeing require further attention in both research and practice in local, international, and other country-specific contexts.

## Authorship Contribution Statement

Magdalena Zadworna contributed to conceptualization, investigation, project administration; resources, formal analysis, validation, writing—original draft. Karolina Kossakowska contributed to methodology, investigation, formal analysis, visualization, data curation, writing—original draft. Tyler Renshaw contributed to supervision, validation, writing—review and editing.

## Data Availability

The datasets analyzed for the current study are not publicly available but are available from the corresponding author on reasonable request.

## Appendix 1

See Table A1.

**Table A1:** The results of confirmatory factor analysis

Factors	Item No	*R*^*2*^	*β*	*B*	*SE*	*CR*
Factor 1 (JLS)	1	.47	.69	1.00		
	5	.58	.76	1.18	.07	18.10 ***
	9	.29	.54	.99	.07	13.49 ***
	13	.49	.70	1.12	.07	17.04 ***
Factor 2 (SCS)	2	.34	.58	1.00		
	6	.39	.62	1.10	.09	12.11***
	10	.43	.66	1.04	.08	12.44***
	14	.42	.65	1.04	.09	12.33***
Factor 3 (APS)	3	.33	.58	1.00		
	7	.39	.63	1.00	.07	13.52 ***
	11	.58	.76	1.23	.08	15.16 ***
	15	.38	.61	1.11	.08	13.28 ***
Factor 4 (AES)	4	.67	.82	1.00		
	8	.71	.84	1.06	.04	24.95 ***
	12	.29	.54	.64	.04	15.28 ***
	16	.61	.78	.92	.04	23.20 ***

*R*^*2*^ – determination coefficient; *β* – standardized regression coefficient; *B* – non-standardized regression coefficient; *SE* – non-standardized regression coefficients error; *CR* – critical ratio; *** indicates *p* < .001

JLS—Joy of Learning Scale; SCS—School Connectedness Scale; APD—Educational Purpose Scale; AES—Academic Efficacy Scale

## Appendix 2

See Table A2.

**Table A2:** Pearson’s *r* Correlation Coefficients for the Analysis of Concurrent and Divergent Validity of the SSWQ Against the KIDSCREEN-27 and the STAIC According to Grade Level

		5th grade^1^			6th grade^1^			7th grade^1^			8th grade^1^		
Variable	*Range**	*M*	*SD*	*r*	*M*	*SD*	*r*	*M*	*SD*	*r*	*M*	*SD*	*r*
KIDSCREEN-27													
Physical Wellbeing	5–25	19.33	3.5	.19*	18.98	3.7	.27*	17.88	4.2	.39**	17.24	3.9	.15*
Psychological Wellbeing	7–35	26.18	5.3	.42**	24.35	5.4	.16	22.69	6.2	.40**	22.84	5.6	.36**
Autonomy Parents	7–35	25.24	5.6	.43**	25.26	6.0	.21*	24.27	6.3	.35**	24.23	5.1	.31**
Peers Social Support	4–20	14.06	3.9	.27**	13.69	4.4	.01	13.82	4.3	.31**	13.49	3.7	.25**
School Environment	4–20	12.79	3.3	.74**	11.73	3.3	.50**	12.15	3.4	.66**	12.12	3.3	.66**
General Index	51–131	97.78	15.9	.54**	94.20	16.7	.29**	91.08	18.2	.54**	90.81	14.3	.48**
STAIC													
State scale	20–74	32.83	9.5	−.32**	32.16	9.4	−.17	34.92	10.1	−.23**	35.69	9.3	−.28**
Trait scale	20–57	33.93	8.0	−.11	32.87	8.4	.02	36.57	8.9	−.12	37.44	7.9	−.11
